# Success

**Published:** 1898-10

**Authors:** Geo. J. Manson


ARTICLE VI.

### SUCCESS.

To succeed in any undertaking there must be an intense longing to realize a definitely conceived ideal which seems to be worthy of any sacrifice. Taine says that "success in life depends on knowing how to be patient, how to endure drudgery, how to un make and re-make, how to recognize and continue without allowing the tide of anger or the flight of imagination to arrest or divert the daily effort."

That a brilliant man cannot succeed by simply relying on the work of his talents is shown by the continuousness of effort displayed by famous men on some of their works. Gray spent seven years in perfecting his "Elegy" which can be read in seven minutes. Prescott worked twenty years in the libraries of Europe collecting material for his Spanish histories, and a large portion of that time he was stricken with blindness so that he had to make use of the eyes of another. Gibbon re-wrote his "Memoirs" nine times, Newton one of his philosophical works fifteen, and Addison his essays twenty times. Spinoza and Buckle each spent twenty years in carefully forming and maturing their judgment before they published their systems of thought. Balzac, before he commenced a work of fiction, wandered week after week up and down the streets of Paris. studying phases of character and prying into the different modes of life. Then he excluded himselt from society for months at a time perfecting his plot and writing the work. He read the proof two or three times and made many corrections. Dr. Harvey spent eight, Dr. Jenner twenty, and Sir Charles Bell forty years in maturing their three famed discoveries in medical science. Titian painted daily on one picture for seven years and for eight on another.

A man can properly consider himself successful if he accomplishes the best results to be attained in his own particular sphere. As an editorial writer in the New York *Sun* said, not long since ; "The truth is, that man is most successful who best and most fully puts to useful service all his powers and faculties, who finds and utilizes the opportunity for their employment; or, in other words, gets into the place which he is best fitted to fill," whether it is in the ordinary walk of life, or in a larger and higher sphere, he is equally successful, providing he is putting forth all the energy and making use of all the capacity for work within him. Thousands of men fail because they are trying to do the work for which they are not fitted. Stentor was not a great man, but his name has been handed down through history as the most successful herald in giving his commands to the Grecian army, Homer calling him "great-hearted, brazen-voiced Stentor, accustomed to shout as loud as fifty other men."

It is necessary that a man shall like his work, or at least like it as well as he can like work of any kind, because he must enter into it with enthusiasm. "Without enthusiasm," said Montalembert, "your life will be a blank, and success will never attend it. Enthusiasm is the one secret of success. It blinds us to the criticisms of the world, which so often dampen our very earliest efforts; it makes us alive to one single object, that which we are working at—and fills us not with the desire only, but with the resolve, of doing well whatever is occupying our attention." Dr. Arnold, of Rugby, used to say that the main difference between men in life is simply one of energy; but, after all, energy is of no use if it is misdirected. A man engaged in an occupation for which he is unfitted may exhaust all his energy in an effort to bring about satisfactory results, Then, again, there is a distinction between energy and activity. Some men are busy enough, but their efforts, having been misdirected, even in a vocation for which they are fitted, have been simply a waste of activity. A marksman may fire all day at a target, but if he does not take a good aim, his time and powder are wasted. Most men mix a pound of ambition with a grain of energy, and then wonder that the world refuses to admire the mixture.

Even in the spiritual realm a man cannot attain to lofty heights without energy. "No man," says Cecil, ever found a happy life by chance, or yawned it into being with a wish. Even the Kingdom of Heaven suffereth violence and the violent only take it by force."

Sir Andrew Clark, President of the Royal College of Physicians, once said that he believed that every man's success was within himself, and must come out of himself, that it must be seriously in earnest, acting with singleness of heart and purpose, and must learn to love work through practicing the habit of working until "at last you will not only abhor idleness, but you will have no happiness out of the work which then you are constrained from love to do."

Every important business plan must be properly thought out before action is taken. You may jump at a conclusion, but you cannot spring into a success. The aphorism of the Duke of Wellington ought never to be forgotten, that success can only be attained by tracing every part of every operation from its origin to its concluding point. After all, Davy Crockett's saying would seem to be the golden rule of business: "Be sure you're right, then go ahead," Old Commodore Vanderbilt, being asked one day what he considered to be the secret of success in business, replied: "Secret? There is no secret about it. All you have to do is to attend to your business, and go ahead."

A man may have every qualification for success and yet fail if he does not take advantage of opportunities. He must be wise enough to recognize, and quick enough to profit by them. A famous sculptor once showed a visitor the treasures of his studio. In it were many mythical gods. One particularly attracted the visitor's attention. The face was concealed by being covered with hair, and there were wings to each foot,

"What is his name ?" said the spectator.

"Opportunity," was the reply.

"Why is his face hidden ?"

"Because men seldom know him when he comes to them."

"Why has he wings upon his feet ?"

"Because he is soon gone, and once gone he cannot be overtaken."

Another useful qualification for success is tact. Without it the best intentioned are forever meeting with obstacles and hindrances, while he who possesses it finds friends who are ever ready to help him on his way. It is the attribute which hides one's motive, as the bitter components of a pill are secreted within their environment of sugar. The boor speaks his mind, but the tactician minds his "speak."

There is nothing very striking in the advice of business men as to how ordinary mortals shall attain fortune. The maxims of the elder Baron Rothschild were to attend to details; be prompt; take time to consider, and then decide quickly; dare to go forward; make no useless acquaintances; shun liquor; employ your time well; do not reckon on chance. "Then work hard and you will be certain to succeed:" Not at all certain, because no notice is here taken of adaptability to and love of the vocation, opportunity, tact, good judgment and enthusiasm, all of them more important factors than those mentioned for the attainment of good fortune. Andrew Carnegie thinks that the free dangers in a young man's path are the addiction to liquor, speculation, and endorsing. The most important statement he makes in connection with the subject is, "It is not capital your seniors require; it is the man who has proved that he has the business habits which make capital."

A pithy explanation of the success of quack doctors and patent medicines is given by one who ought to know. A quack in immense practice in London once consulted the great physician Abernethy for some personal ailment. Curious to know the secret of his success in attracting clients, the surgeon asked him if he knew why he had so many more patients than any regular physician. Drawing Abernethy to the window, he pointed to the surging crowd in the street below, and asked :

"Doctor, of every one hundred persons who pass this window, how many do you suppose are educated people, capable of appreciating study and cultivate in others ?"

"Perhaps ten," answered the famous surgeon.

"Exactly," rejoined the charlatan, "Well, you get those ten; the rest come to me."

If we can discover definitely why men fail in business, we will be far on the road toward learning how they can succeed. Here, again, it is difficult to lay down absolute rules that will apply to all. Men fail simply because they lack one or more of the numerous elements that go to make up success. A leading New York merchant who has given years of careful study to the cause of so many failures among the commercial classes says : "I think the causes of commercial failures may be classified as follows : Six-tenths ensue from inexperience, extravagance and negligence. Two tenths from natural dis-dishonesty. One-tenth from speculation. One tenth, the unfortunate man. There is not sufficient attention given to the training of men for mercantile life. Every young man intending to follow mercantile pursuits ought to spend some years in preparation in a methodically conducted establishment. He should be trained as to values, how to buy and how to sell, and also as to management, from the picking up of the string from the floor to the banking of his cash. There are instances where men and women of modest pretensions have commenced in a small way and finally succeeded; but their training comes with the development of their business. They know not only how to make a little money, but also how to save what they make."—GEO. J. MANSON, in success.

